# Supplementary material for: Room temperature electrically pumped topological insulator lasers
Source: Nat Commun. 2021 Jun 8;12:3434. doi: 10.1038/s41467-021-23718-4 (PMC8187422; doi:10.1038/s41467-021-23718-4)
Supplement: Supplementary file 1 — Supplementary Information [file 41467_2021_23718_MOESM1_ESM.pdf]

Supplementary Information for

Room temperature electrically pumped topological insulator lasers

Jae-Hyuck Choi[1], William E. Hayenga[1,2], Yuzhou G. N. Liu[1], Midya Parto[2], Babak Bahari[1], Demetrios N. Christodoulides[2], and Mercedeh Khajavikhan[1,2*]

[1]Ming Hsieh Department of Electrical and Computer Engineering, University of Southern California, Los Angeles, California 90089, USA.

[2]CREOL, The College of Optics & Photonics, University of Central Florida, Orlando, Florida 32816–2700, USA.

*email: khajavik@usc.edu

Note 1. Wafer structure and fabrications procedure

The electrically pumped topological insulator laser is based on an InGaAsP/InP epitaxial structure, grown on an undoped InP substrate by molecular beam epitaxy (MBE) (Table S1). The gain region consists of 320 nm of undoped multiple quantum wells, sandwiched between an n-doped (with a thickness of 550 nm) and a p-doped (with a thickness of 700 nm) InP layers. The multiple quantum well structure is comprised of 10 layers of $In_{x=0.564}Ga_{1-x}As_{y=0.933}P_{1-y}$ (each with a thickness of 10 nm) placed between 11 layers of $In_{x=0.737}Ga_{1-x}As_{y=0.569}P_{1-y}$ (each with a thickness of 20 nm). Highly doped n-InGaAs and p-InGaAsP layers form the n- and p-contacts, respectively. The doping levels in the p- and n- doped InP layers, in the upper and lower sides of the gain region, gradually increase to reach the highest level at the contact layers. The epitaxial structure, designed by our group, was grown by OEpic Semiconductors Inc. [1].

Material	Loop	Thickness (nm)	Dopant	Comment
InGaAs	1	125	Zn (p ~2E19 cm^{-3})	n-contact layer
p-InP	1	350	Zn (p ~5E18 cm^{-3})	Cladding
p-InP	1	350	Zn (p ~1E18 cm^{-3})	
$In_{x=0.737}Ga_{1-x}As_{y=0.569}P_{1-y}$	1	20	Undoped	QW barriers
$In_{x=0.564}Ga_{1-x}As_{y=0.933}P_{1-y}$	10	10	Undoped	QWs
$In_{x=0.737}Ga_{1-x}As_{y=0.569}P_{1-y}$	10	20	Undoped	QW barriers
n-InP	1	300	Si (n ~1E18 cm^{-3})	Cladding
n-InP	1	250	Si (n ~5E18 cm^{-3})	
InGaAsP	1	125	Si (n ~2E19 cm^{-3})	p-contact layer
InP	1	1000	Si (n ~2E19 cm^{-3})	Buffer
InP substrate			Undoped	

Table S1. Detailed wafer structure with doping levels and layer thicknesses.

In order to fabricate the electrically pumped topological insulator lasers (Figure S2), first, the pattern of the structure is defined onto FOx-16 e-beam resist by high-resolution e-beam lithography (Fig. S2a), then it is transferred into the wafer by a reactive ion etching (RIE) process using H_2:CH_4:Ar ($10:40:7$ sccm) plasma (Fig. S2b). Figure S3 presents the scanning electron microscope (SEM) image of the array at this stage of fabrication. Next, a 240 nm thick silicon nitride (Si_3N_4) film is deposited by plasma-enhanced chemical vapor deposition (PECVD) (Fig. S2c). To access the underlying highly doped InGaAsP layer, the cathode-electrode pattern is defined by photolithography, followed by a sequential dry etching of the Si_3N_4 layer, and wet etching of the remaining InP n-doped layer. Finally, the cathode-electrode is formed by photolithography and metal deposition of Ni/AuGe/Au ($5/75/300$ nm) (Fig. S2d). After photoresist liftoff, to planarize the surface before depositing the anode-electrode, the surface is spin-coated by an optically transparent polymer material (benzocyclobutene, BCB). Even though the BCB polymer is expected to provide a uniform planarization layer on the sample, in practice it tends to show some non-uniformities. The presence of these non-uniformities makes it difficult to properly pump the lasers, thus causing issues with overheating. In some cases, like on top of the gratings, we noticed that it is even more difficult to fully etch down the BCB and reach the contact layers for pumping. As a result, in our samples, the gratings remained fully unpumped. These issues may be addressed by developing new BCB spinning techniques and using thicker metal layers for electrodes. The thickness of the BCB layer was reduced by O_2:CF_4 ($10:5$ sccm) plasma etching until the top of the topological lattice structure is extruded (Fig. S2e). The anode-electrode area is then defined by photolithography. To reach the InGaAs p-contact layer, the additional Si_3N_4 layer and FOx-16 are removed from the top of the device using an RIE dry-etching process followed by a BOE wet-etching step (Fig. S2f). The anode-electrode metal film Ti/Au ($15/300$ nm) is then deposited using thermal evaporation (Fig. S2g). After liftoff, a rapid thermal

annealing (RTA) is performed at a temperature of 380 ℃ for 30 s to fuse the metal and reduce the resistance of the contact (Fig. S2h). Lastly, the sample is mounted on a header and is wire-bonded for optical and electrical characterizations (Fig. S2i).

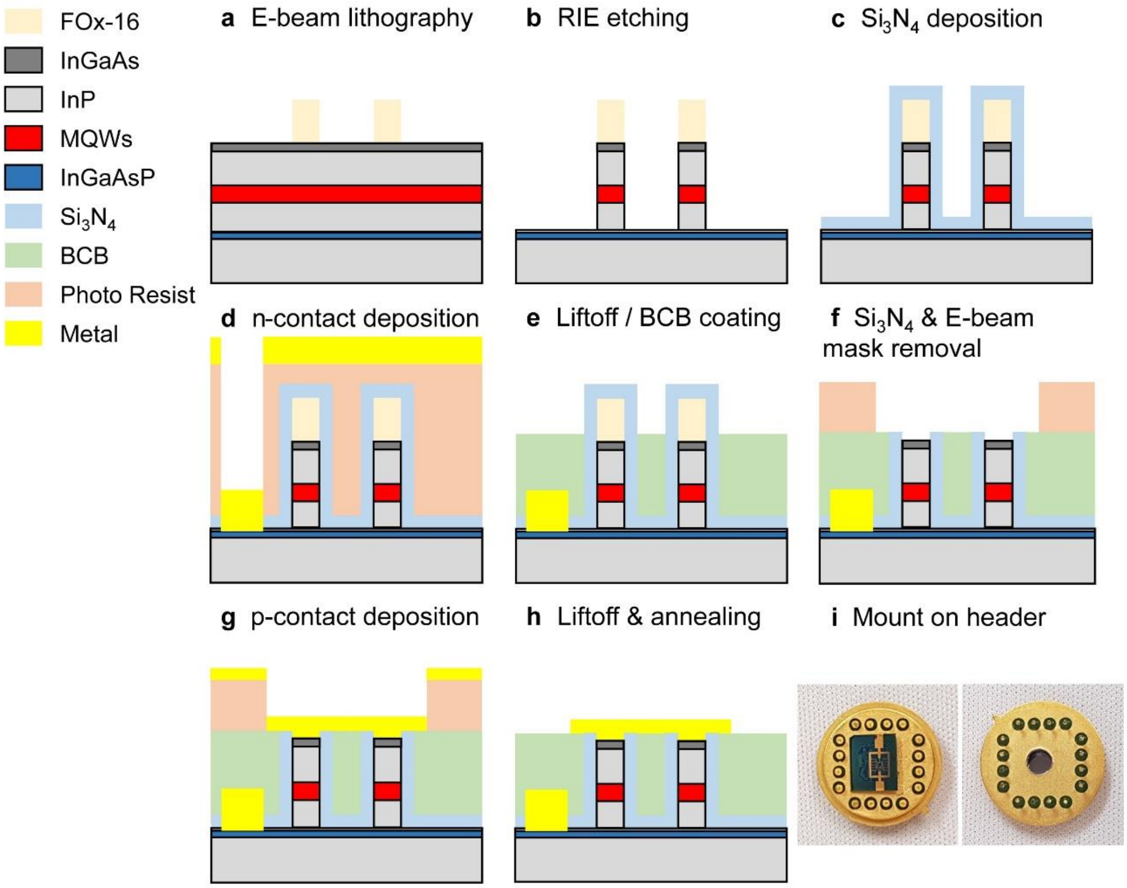

Figure S2. Fabrication steps involved in realizing electrically pumped topological insulator lasers. **a** The lattice structure is defined on a FOx-16 resist using e-beam lithography. **b** Pattern is transferred to the wafer using RIE etching. **c** Silicon Nitride is deposited using PECVD. **d** Cathode-electrode is fabricated on the InGaAsP contact layer. **e** After liftoff, BCB is spin-coated and dry etched. **f** The excess Silicon Nitride and FOx-16 are removed. **g** Anode-electrode is deposited on the InGaAs p-contact layer. **h** Liftoff and annealing are performed. **i** The sample is mounted on the header and wire-bonded to the pins.

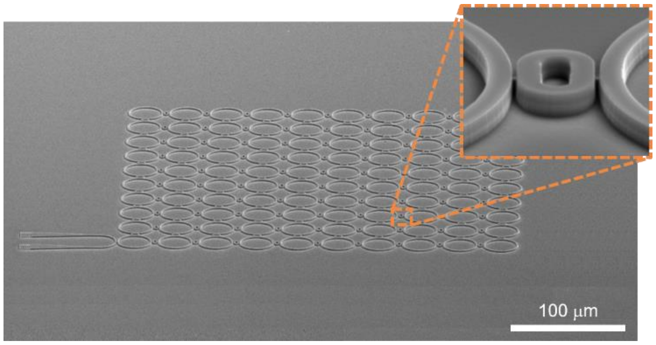

Figure S3. Scanning electron microscope (SEM) image of a fabricated topological insulator laser array. A 10 × 10 resonator network is connected via link structures. Inset: An image of a link resonator and its adjacent ring resonators. A 200 nm gap exists between the ring resonator and the off-resonant link. The etched depth of the structure is 1650 nm. The gain region can be identified in the middle of the structure.

Note 2. Electromagnetic simulations

In order to identify the lasing mode in the standalone ring resonators, finite element method (FEM) simulations are performed using COMSOL Multiphysics package. The microring resonator used in this simulation has a radius of 15 μm and a width of 1.4 μm. The cross section of the microring is composed of InGaAsP multiple quantum wells sandwiched between InP cladding layers and the sidewall is covered by a 240 nm thick Si_3N_4 film. The structure is covered by BCB on the side and air on top. To find the eigenmodes in the microring resonators, a 2-D axisymmetric simulation is performed using eigenmode solver module (Fig. S4a). Here, the refractive index of the gain region (MQWs) is 3.4 [2] and the refractive index of InP is 3.14 [3]. Figure S4a shows the fundamental transverse electric mode (TE_0), primarily confined in the InGaAsP multiple quantum wells region. With this cross section profile, the effective index of the TE_0 mode is $n_{\mathrm{eff}} = 3.2$. This effective index is used to perform the 2-D simulation of a ring resonator with a radius of 15 μm (Fig. S4b).

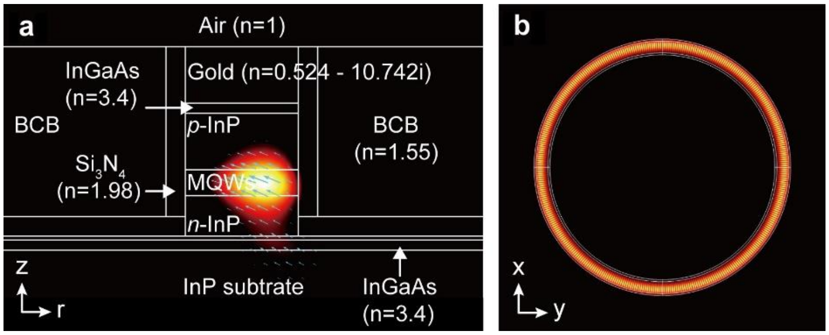

Figure S4. Lasing mode in a standalone ring resonator. **a** Cross section of the electric field intensity distribution shows the confined mode in the multiple quantum well gain region. The effective index of the mode is 3.2. **b** Electric field intensity distribution of the fundamental transverse electric (TE_0) mode inside the 2-D ring resonator.

Note 3. Measuring coupling strength

In our experiments, we characterize the coupling strength between two site ring resonators. The coupling is mediated by a link resonator [4]. To do this, we fabricate a basic building block of the structure, comprising of two site rings and one off-resonant link on the electrically pumped wafer as shown in Note 1. The gap size between the rings and link is 200 nm and the structural parameters of the elements are the same as the topological insulator laser reported in the main manuscript. Under optical pumping, a frequency splitting of ~31 GHz (0.245 nm in wavelength) is observed in the emission spectrum which indicates a coupling strength of $\kappa = \Delta\nu/2 = 15.5$ GHz.

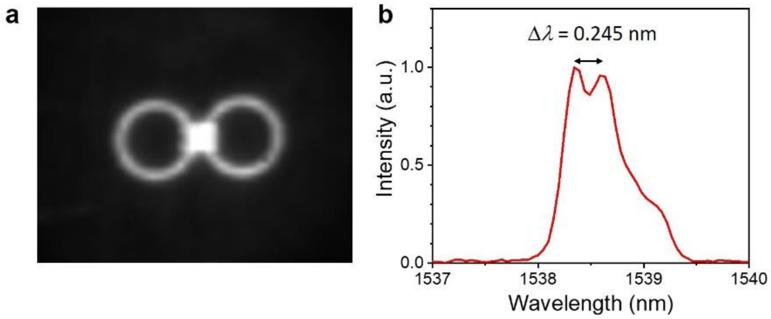

Figure S5. **a** The captured emission profile image from two ring resonators coupled via a link resonator when all the elements are optically pumped. **b** The spectrum shows a frequency splitting of 0.245 nm corresponding to a coupling strength of 15.5 GHz.

Note 4. Characterization setup

Figure S6 depicts the schematic of a dual function measurement station, designed for simultaneous micro-electroluminescence (μ-EL) and micro-photoluminescence (μ-PL) characterizations. For μ-EL characterization, the samples are examined under pulsed pumping, using a current driver (ILS Lightwave LDP-3840B) with a pulse width of 300 ns (100 ns) and a period of 50 μs when all (one) anode electrodes are connected. A $10\times$ microscope objective lens with a numerical aperture of 0.26 is used to collect the emission from the topological insulator lasers. The surface of the sample is imaged by two cascaded 4-f imaging systems into a NIR camera (Xenics Inc.). A broadband ASE source that passes through a rotating ground diffusing glass is used to illuminate the sample surface (as an incoherent source). A notch filter is placed in the path of emission to attenuate the pump beam. The emission spectra are obtained using a spectrometer (Princeton Instruments Acton SP2300) with an attached linear array detector (Princeton Instruments OMA V).

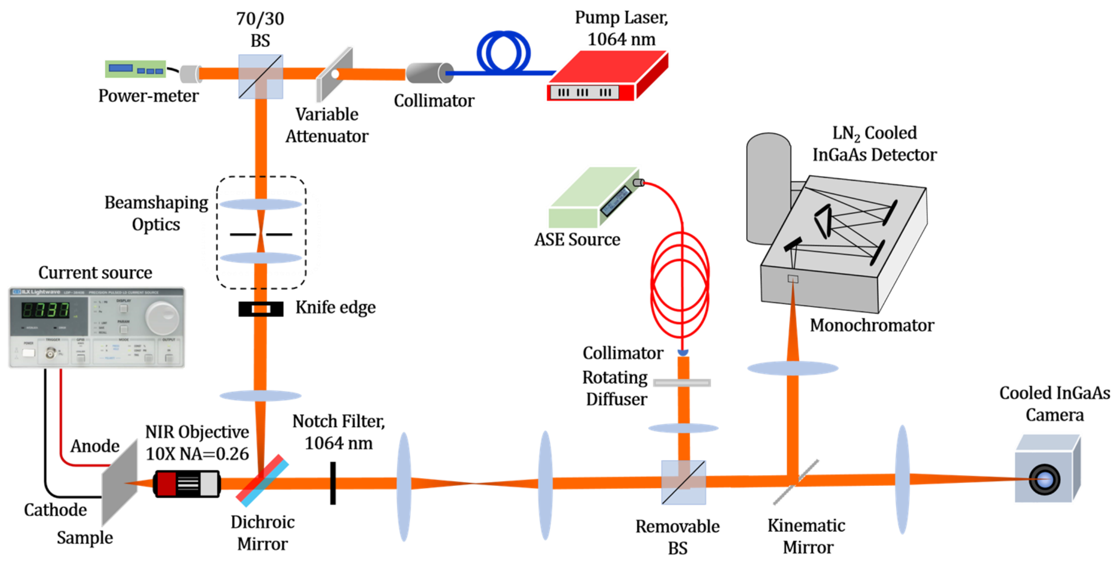

Figure S6. Schematic of the micro-electroluminescence and micro-photoluminescence characterization setup. The topological insulator laser is pumped by a pulsed current source. The emission is collected by a 10 × objective lens and directed into an NIR detector array for spectral measurements or to a NIR camera for intensity profile observation.

The μ-PL characterization part of the setup is utilized to examine the existence of the topological edge modes under optical pumping condition. In this setup a pump laser (SPI fiber laser) beam at a wavelength of 1064 nm is used with a duration of 15 ns and duty cycle of 0.4% (Fig. S6). The 10 × microscope objective is used to project the pump beam on the ring resonator and also serves to collect the emission. A square-shaped metallic intensity mask and a knife edge are placed at the object plane of the imaging system. The shadow of the metal mask and the knife edge are imaged on the sample surface to provide the desired pump profile as shown in Fig. S7.

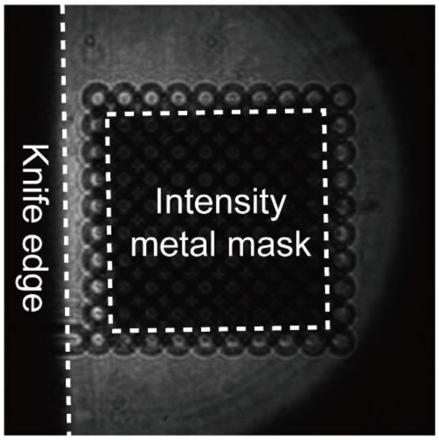

Figure S7. Optical pump profile provides gain along the perimeter of the array to promote the lasing of the topological edge mode.

Note 5. Measured lasing spectra from electrically pumped topological insulator laser at various edge elements

Figure S8 shows the collected emission from sites L2, L4, L5 and R6 when all the perimeter elements are electrically pumped. In all these cases, the lasing peak was observed at a wavelength of 1503 nm (similar to what was measured at R3). Also, as can be seen in these plots, the spectra all around the cavity exhibit single mode lasing behavior.

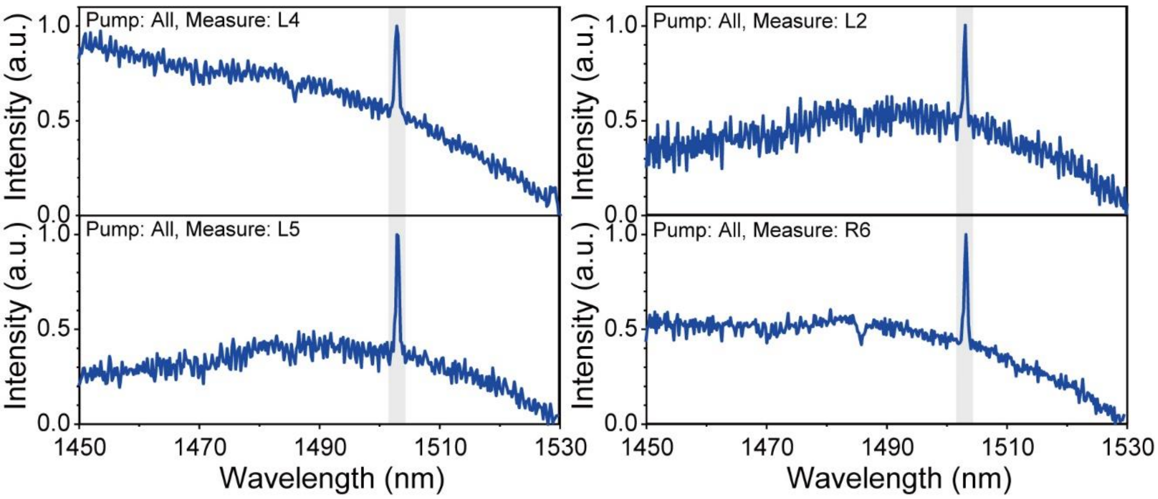

Figure S8. Measured emission spectra under electrical pumping at various sites (L2, L4, L5, and R6).

Note 6. Measured I-V curves of topological insulator laser

Figure S9 shows I-V curves of a topological insulator laser when all the electrodes are connected (Figure S9a), and only one electrode is connected (Figure S9b).

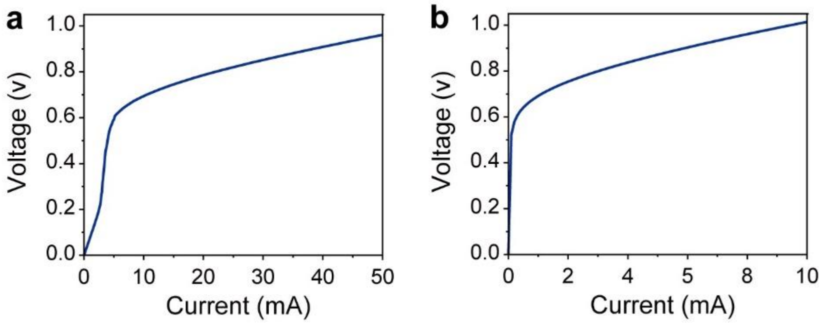

Figure S9. Measured I-V curves from topological insulator lasers when **a** all the electrodes are connected, and **b** only one electrode is connected.

Note 7. Comparison of topologically trivial and non-trivial lattices

To compare the lasing properties of the topologically trivial and non-trivial lattices, we characterize a topologically trivial lattice that has no position shift of the link resonators ($\Delta x = 0$). This corresponds to the $\alpha = 0$ case. Unlike the emission spectra collected from the topological insulator laser array, when the perimeter of the trivial lattice is pumped, multiple longitudinal lasing modes appear in the spectrum. Figure S10 compares the lasing spectra of topologically trivial and non-trivial lasers when the perimeters of the arrays are pumped at the same level. Please notice that for demonstration purposes the spectrum of trivial lattice is scaled up by a factor of 3.

The measurements are performed on samples of electrically pumped devices before the deposition of the electrodes. After depositing the metal electrodes, the trivial lattice shows no lasing, whereas the topological array lased as shown before.

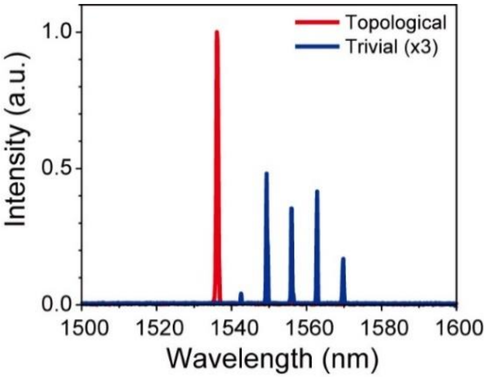

Figure S10. Measured emission spectra of topologically trivial and non-trivial laser arrays under the same pump level.

Note 8. Robustness of topological insulator laser to disorder

In order to investigate the robustness properties of the topological insulator lasers to disorder, we examine a topological insulator structure with missing site rings (un-pumped) on the perimeter elements (Fig. S11). When all the perimeter elements of the array are electrically pumped via the twelve electrodes, a single mode lasing peak is observed from different pumping sites. This result shows that our electrically pumped topological insulator lasers exhibit topological protection even if some of site rings are not pumped or missed.

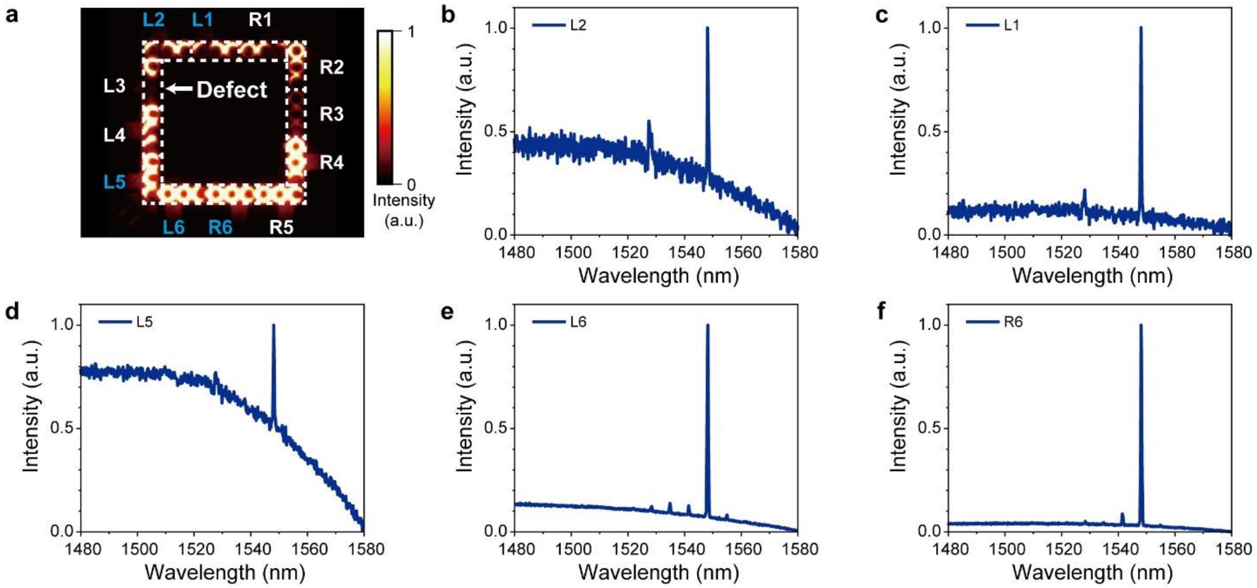

Figure S11. **a** Intensity profile images of topological insulator laser when two site rings of the topological edge element are not electrically pumped. **b-f** Measured electroluminescent spectra at each different site.

Note 9. Coherence measurement

To confirm that our topological laser elements emit in a coherent fashion**,** the interference patterns formed by the emission from various sites along the periphery of the array are measured. In order to perform this measurement, we modified our measurement setup by adding two branches operating as a Mach-Zehnder interferometer. Figure S12 shows a schematic diagram of this modified setup. Each interferometer arm has a moving iris that can select the emission from a desired site. These emissions are overlapped and the resulting inference fringes are imaged in the camera. Figure S13a shows the measured interference images obtained by overlapping emission from two neighboring sites when the perimeter of the topological insulator laser is electrically pumped. On the other hand, Fig. S13b depicts the interference result from two neighboring elements in the trivial laser under optical pumping- where no fringes could be identified. Unfortunately, interference measurements between larger apart sites were not performed due to the limitation of the field of view in our setup. In order to verify the extension of the coherence across the topological laser array, we repeated the interference measurement for every two consecutive elements on the edge (1&2, 2&3, 3&4, etc.). Figure S14 shows the resulting fringes of this extensive study along one edge. These measurements together with the observed single mode lasing operation can be an indication of a widespread coherence over the entire edge elements in the topological laser array.

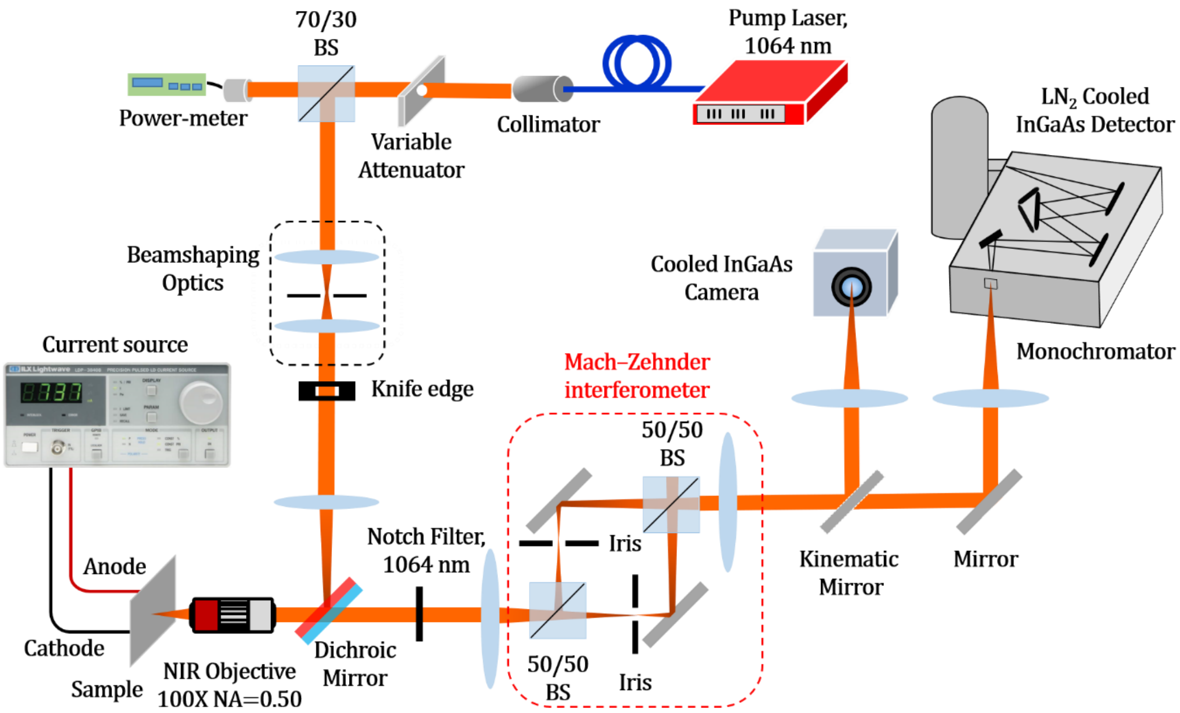

Figure S12. Schematic of the micro-electroluminescence characterization setup with the added Mach-Zehnder interferometer for measuring coherence.

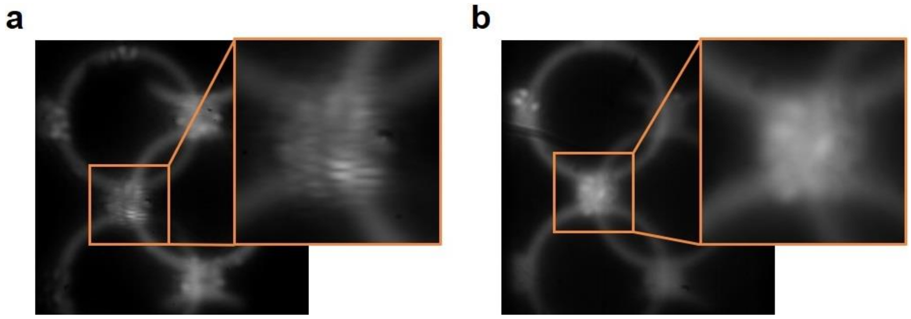

Figure S13. Measured intensity profile of interference fringes when the emission of two different neighboring site rings of the **a** topologically non-trivial and **b** trivial arrays are overlapped. Inset: Magnified interference pattern image. The topological laser shows interference fringes, while no such fringes could be observed in the trivial laser array.

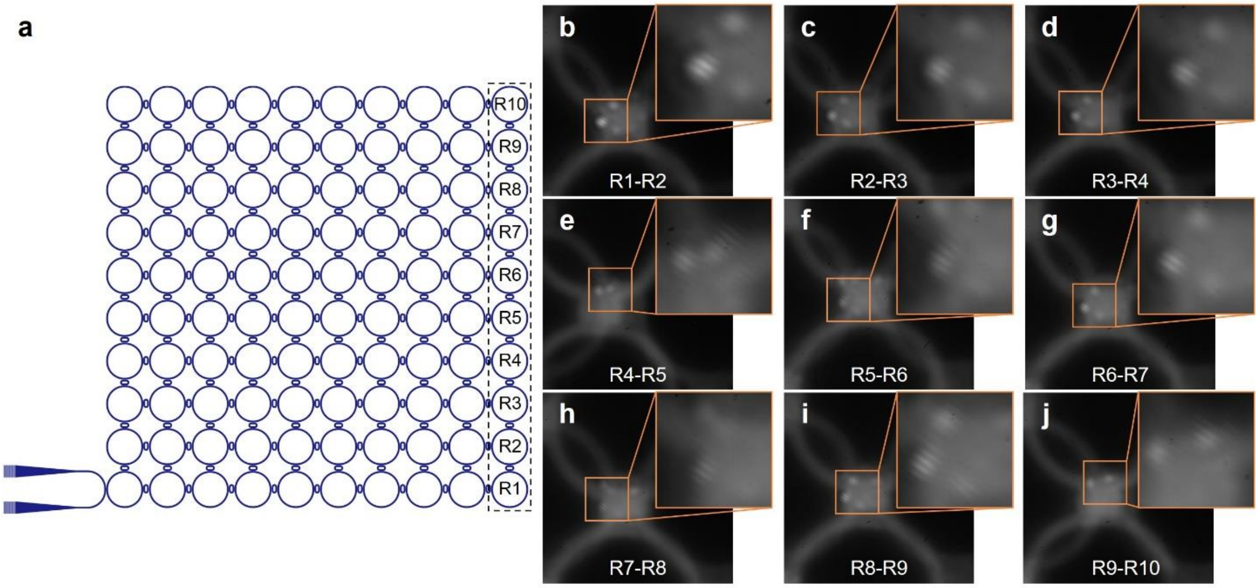

Figure S14. **a** A schematic diagram of the topological laser array showing the location of the overlapped ring elements. **b-j** Measured interference fringes for elements along one edge of the topological laser array. Insets: Magnified interference pattern image.

Note 10. Unidirectional lasing by incorporating S-bends inside the site ring resonators

We should note that our current structure is not designed to lase in a predetermined unidirectional fashion. In quantum spin Hall based topological lasers, pumping can excite both clockwise (CW) and counter clockwise (CCW) modes. While no unidirectionality is expected from passive quantum spin Hall photonic arrangements, the lasers based on this effect tend to show some degree of chirality because of the hole burning mechanism in inhomogenously broadened gain systems. However, since we did not collect the emission from the gratings, we cannot verify if and to what extent the emission is unidirectional. Nevertheless, spontaneous breaking of chirality offers no practical advantage in lasers.

We are currently working on adding S-bends to our electrically pumped topological lasers to enforce predetermined unidirectionality in our lasers. However, adding these constructs, even though possible, requires overcoming several additional challenges. For example, the distance between the S-bends and the rings must be precisely controlled. Also, due to the larger width of the waveguides, the tapering of S-bends becomes more difficult. Finally, one should expect much more non-uniformities of BCB layer in the rings with S-bends, thus causing issues with good quality electrodes.

We designed the electrically pumped lasers with S-bends as shown in Fig. S15, and characterized the laser performance under optical pumping (without depositing the electrode). From the strong emission emerging from one of the grating output couplers, we can confirm that the S-bend design leads to unidirectional operation. As mentioned above, fabricating uniform top electrodes for both rings and S-bends is quite challenging. We are currently working to find a solution for this problem for our future designs.

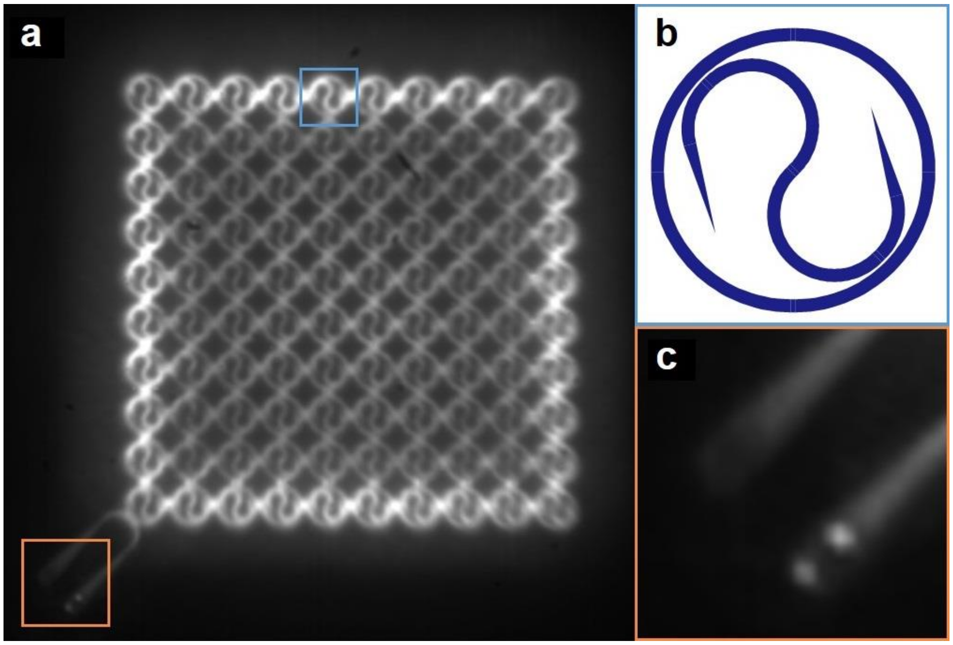

Figure S15. a Measured intensity profile from a topological insulator array fabricated on a wafer suitable for electrical pumping with S-bends inside the ring resonators. The measurement is performed through optical pumping and prior to top electrode deposition. **b** A ring resonator with an S-bend. **c** The magnified image of the grating output couplers. The unidirectionality of the topological edge mode can be confirmed from the strong emission observed at only one of the grating couplers.

Supplementary References:

1. Hodaei, H. et al. Parity-time–symmetric microring lasers. *Science* **346**, 975–978 (2014).
2. Khajavikhan, M. et al. Thresholdless nanoscale coaxial lasers. *Nature* **482**, 204–207 (2012).
3. Adachi, S. Optical dispersion relations for GaP, GaAs, GaSb, InP, InAs, InSb, AlxGa1−xAs, and In1−xGaxAsyP1−y. *Journal of Applied Physics* **66**, 6030-6040 (1989).
4. Bandres, M. A. et al. Topological insulator laser: experiments. *Science* **359**, eaar4005 (2018).
