# Supplementary material for: Room temperature electrically pumped topological insulator lasers
Source: Nat Commun. 2021 Jun 8;12:3434. doi: 10.1038/s41467-021-23718-4 (PMC8187422; doi:10.1038/s41467-021-23718-4)
Supplement: Supplementary file 3 — Lasing Reporting Summary [file 41467_2021_23718_MOESM3_ESM.pdf]

nature research

Corresponding author(s): Mercedeh Khajavikhan

Lasing Reporting Summary

Nature Research wishes to improve the reproducibility of the work that we publish. This form is intended for publication with all accepted papers reporting claims of lasing and provides structure for consistency and transparency in reporting. Some list items might not apply to an individual manuscript, but all fields must be completed for clarity.

For further information on Nature Research policies, including our data availability policy, see Authors & Referees.

Experimental design

Please check: are the following details reported in the manuscript?

1. Threshold

Item	Yes/No	Details
Plots of device output power versus pump power over a wide range of values indicating a clear threshold	[X] Yes [ ] No	Figure 2d, Figure 3d

2. Linewidth narrowing

Item	Yes/No	Details
Plots of spectral power density for the emission at pump powers below, around, and above the lasing threshold, indicating a clear linewidth narrowing at threshold	[X] Yes [ ] No	Figure 2c, Figure 3c
Resolution of the spectrometer used to make spectral measurements	[X] Yes [ ] No	caption of Figure 3

3. Coherent emission

Item	Yes/No	Details
Measurements of the coherence and/or polarization of the emission	[X] Yes [ ] No	Discussion (in the main text), Supplementary Note 9

4. Beam spatial profile

Item	Yes/No	Details
Image and/or measurement of the spatial shape and profile of the emission, showing a well-defined beam above threshold	[X] Yes [ ] No	Figure 2a, Figure 3b

5. Operating conditions

Item	Yes/No	Details
Description of the laser and pumping conditions<br>*Continuous-wave, pulsed, temperature of operation*	[X] Yes [ ] No	Main manuscript page 6 line 11 (optical pumping), page 7 line 10 (electrical pumping)
Threshold values provided as density values (e.g. W cm^{-2} or J cm^{-2}) taking into account the area of the device	[X] Yes [ ] No	Main manuscript page 7 line 1 (optical pumping), page 8 line 1 (electrical pumping)

6. Alternative explanations

Item	Yes/No	Details
Reasoning as to why alternative explanations have been ruled out as responsible for the emission characteristics<br>*e.g. amplified spontaneous, directional scattering; modification of fluorescence spectrum by the cavity*	[X] Yes [ ] No	Characterization and Discussion (in the main text)

7. Theoretical analysis

Item	Yes/No	Details
Theoretical analysis that ensures that the experimental values measured are realistic and reasonable<br>*e.g. laser threshold, linewidth, cavity gain-loss, efficiency*	[X] Yes [ ] No	Design and Fabrication (in the main text)

8. Statistics

Item	Yes/No	Details
Number of devices fabricated and tested	[X] Yes [ ] No	We tested(and fabricated) multiple devices of optical pumping, electrical pumping, defects and S-bend samples.
Statistical analysis of the device performance and lifetime (time to failure)	[ ] Yes [X] No	Does not apply
